# Berberine ameliorates diabetic nephropathy by inhibiting TLR4/NF-κB pathway

**DOI:** 10.1186/s40659-018-0157-8

**Published:** 2018-03-31

**Authors:** Liping Zhu, Jiakai Han, Rongrong Yuan, Lei Xue, Wuyan Pang

**Affiliations:** Department of Endocrinology, Huaihe Hospital of Henan University, No.115 Ximen Street, Gulou District, Kaifeng, 475000 China

**Keywords:** Berberine, Streptozotocin, Diabetic nephropathy, Podocytes, TLR4/NF-κB pathway, Inflammatory response

## Abstract

**Background:**

Diabetic nephropathy (DN) is the leading cause of end-stage renal failure, contributing to severe morbidity and mortality in diabetic patients. Berberine (BBR) has been well characterized to exert renoprotective effects in DN progression. However, the action mechanism of BBR in DN remains to be fully understood.

**Methods:**

The DN rat model was generated by intraperitoneal injection of streptozotocin (STZ, 65 mg/kg body weight) while 30 mM high glucose (HG)-treated podocytes were used as an in vitro DN model. The fasting blood glucose level and ratio of kidney weight to body weight were measured after BBR treatment (50, 100, or 200 mg/kg) in STZ-induced DN rats. The renal injury parameters including 24-h urinary protein, blood urea nitrogen and serum creatinine were assessed. qRT-PCR was performed to detect the transcript amounts of inflammatory factors. The concentrations of inflammatory factors were evaluated by ELISA kits. Western blot analysis was conducted to measure the amounts of TLR4/NF-κB-related proteins. The apoptotic rate of podocytes was analyzed by flow cytometry using Annexin V/propidium iodide.

**Results:**

Berberine reduced renal injury in STZ-induced DN rat model, as evidenced by the decrease in fasting blood glucose, ratio of kidney weight to body weight, 24-h urinary protein, serum creatinine, and blood urine nitrogen. BBR attenuated the systemic and renal cortex inflammatory response and inhibited TLR4/NF-κB pathway in STZ-induced DN rats and HG-induced podocytes. Also, HG-induced apoptosis of podocytes was lowered by BBR administration. Furthermore, blockade of TLR4/NF-κB pathway by resatorvid (TAK-242) or pyrrolidine dithiocarbamate aggravated the inhibitory effect of BBR on HG-induced inflammatory response and apoptosis in podocytes.

**Conclusions:**

Berberine ameliorated DN through relieving STZ-induced renal injury, inflammatory response, and podocyte HG-induced apoptosis via inactivating TLR4/NF-κB pathway.

## Background

Diabetic nephropathy (DN) is a major complication in patients with either type 1 or type 2 diabetes mellitus and one of the leading causes of end-stage renal failure, contributing to severe morbidity and mortality in diabetic patients [1]. It is characterized by microalbuminuria, glomerular and tubular epithelial hypertrophy, excessive accumulation of extracellular matrix (ECM) protein, thickening of glomerular and tubular basement membranes, which eventually results in the failure of renal function [2]. Accumulating evidence has demonstrated that metabolic and hemodynamic factors, including hyperglycemia, transforming growth factor-β1 (TGF-β1) and angiotensin II are implicated in the development of DN [3]. Among these factors, hyperglycemia is currently regarded as the key initiating factor in the progression of DN [4]. In addition, it is believed that inflammatory processes are also able to accelerate the development and progression of DN [5]. According to the previous studies, DN was accompanied by the increased amount of pro-inflammatory cytokines and chemokines, including tumor necrosis factor-α (TNF-α), interleukin (IL)-1β and IL-6 [6]. Podocytes have been recognized as critical regulators of glomerular injury and reduction in podocytes number mediated by apoptosis are associated with the pathogenesis of DN [7, 8]. Although extensive researches have been done in clarifying the pathogenesis of DN, it still remains a challenging task to develop novel and effective therapeutic strategies in the treatment of DN.

Berberine (BBR), an isoquinoline alkaloid isolated from *Coptidis rhizome*, *Cortex phellodendri,* and *Berberis vulgaris*, has been extensively used for long as an oriental medicine to treat gastroenteritis and secretory diarrhea [9–12]. Increasing evidence has exhibited that BBR has a wide range of pharmacological activities, for instance, anti-oxidant stress, anti-inflammatory, anti-tumor, anti-microbial and anti-fibrosis effects, suggesting the clinical and research value of BBR [13]. Furthermore, BBR has been well characterized to exert renoprotective effects in DN progression [14, 15]. However, the underlying mechanism of the renoprotective effect of BBR on DN remains to be further explored.

Toll-like receptors (TLRs) are a conserved family of pattern recognition receptors in the innate immune system that activate downstream inflammatory signaling pathways in response to exogenous microbial pathogens [16]. Activation TLRs signal was associated with the activation of nuclear factor kappa B (NF-κB) activity and consequent increased release of pro-inflammatory cytokines and chemokines such as IL-6, monocyte chemotactic protein-1 (MCP-1), and IL-1β, in turn initiating local inflammation and leukocyte accumulation [17, 18]. NF-κB, a downstream effector of TLR4 signaling pathway, is a ubiquitous and important nuclear transcription factor which mediates several inflammatory processes [19]. There is striking evidence that activation of NF-κB plays a critical role in renal inflammation and fibrosis of the progression of DN [17]. Among all TLRs, TLR4 has been reported to be implicated in the pathogenesis of acute kidney injury, chronic kidney diseases, and the occurrence of DN [20].

In the present study, we used streptozotocin (STZ)-induced in vivo model of DN and high glucose (HG)-induced podocytes as an in vitro model to investigate the protective effect of BBR on DN and its possible molecular basis. Our study demonstrated that BBR could reduce streptozotocin (STZ)-induced renal injury and inflammatory response, and podocyte apoptosis by inhibiting the TLR4/NF-κB pathway.

## Methods

### Animal models and treatment protocols

The animal study was approved by the Experimental Animal Ethical Committee of Huaihe Hospital of Henan University and performed in accordance with the National Institutes of Health Guide for the Care and Use of Laboratory Animals. Male Sprague–Dawley rats (weighing 250 ± 20 g) were purchased from Shanghai Science Academy Animal Center (Shanghai, China) and housed in the standard laboratory conditions. All rats were allowed free access to food and water ad libitum at a controlled temperature room with a constant 12-h light/dark cycle. After a week of adaptive feeding, these rats were randomly divided into 5 groups (n = 10/group): NC group, DN group, DN + BBR (50 mg/kg), DN + BBR (100 mg/kg), and DN + BBR (200 mg/kg). Rats in DN group, DN + BBR (50 mg/kg), DN + BBR (100 mg/kg), and DN + BBR (200 mg/kg) were intraperitoneally injected with 65 mg/kg streptozotocin (STZ, Sigma-Aldrich, St. Louis, MO, USA) dissolved in a 0.1 mM chilled citrate–phosphate buffer (pH 4.5) to induce diabetes [21]. The rats in NC group were injected with chilled citrate–phosphate buffer (0.1 mM, pH 4.4). Control rats in DN group received an equal amount of citrate–phosphate buffer alone by intraperitoneal injection. When the fasting blood glucose levels from tail vein of STZ-induce diabetic rats were over 16.7 mM at 5 days after STZ injection, these rats were considered as diabetes. One week later, the diabetic rats in DN + BBR (50 mg/kg), DN + BBR (100 mg/kg), and DN + BBR (200 mg/kg) were orally treated with BBR dissolved in 0.5% carboxymethyl cellulose at a dose of 50, 100 or 200 mg/kg every day, respectively. The volume of 0.5% carboxymethyl cellulose to dissolve the different doses of BBR (50, 100, and 200 mg/kg) was 4, 2, and 1 ml, respectively. Meanwhile, the rats in NC group and DN group were gavaged with the same volume of 0.5% carboxymethyl cellulose. The fasting blood glucose and body weight were measured every 2 weeks for 8 weeks. The rats were sacrificed at 8 weeks after BBR treatment. Blood samples were collected, and the serum was separated by centrifugation and stored at − 80 °C until analysis. Meanwhile, right kidney samples were rapidly excised, weighted, and stored at − 80 °C until analysis. The ratio of kidney weight to body weight was considered as kidney weight index.

### Biochemical analysis

At the end of the experiment, the animals were maintained in metabolic cages for 24 h to harvest urine for assessing 24-h urinary protein with an enzyme-linked immunosorbent assay (ELISA) kit (Runyu Biotechnology Co., Shanghai, China). The fasting blood glucose level was determined based on the glucose oxidase-catalyzed reaction (chemistry analyzer; Auto Analyzer Quik-Lab, Ames, Spain). To assess renal function, blood urea nitrogen and serum creatinine in the serum of blood samples were measured using an automatic biochemistry analyzer (Hitachi, Tokyo, Japan).

### Determination of IL-1β, IL-6, and MCP-1 levels

Renal corticals were homogenized and centrifuged at 9000×*g* for 30 min at 4 °C. The levels of proinflammatory cytokines in kidney homogenate and serum, including IL-1β, IL-6, and MCP-1, were determined using commercially acquired ELISA kits (Abcam Inc., Cambridge, MA, USA).

### Cell culture and treatment

Conditionally immortalized mouse podocytes were purchased from Yubo Bio-Technique Co. Ltd (Shanghai, China) and cultured in RPMI 1640 medium (Hyclone, Logan, UT, USA) supplemented with 10% fetal bovine serum (FBS; Hyclone), 100 U/ml penicillin/streptomycin, 5.6 mM glucose (Dingguo Changsheng Biotechnology Co., Ltd., Beijing, China) and 10 U/ml recombinant mouse interferon-γ (IFNγ; Pepro Technology, Rocky Hill, NJ, USA) at 33 °C in a 5% CO_2_ humidified incubator. To investigate the effect of BBR on DN, podocytes were pre-treated with 30 mM high glucose (HG) for 24 h prior to treatment with BBR at a dose of 10, 30 or 90 μM for 24 h. In some experiment, podocytes were pre-treated with 30 mM HG in the presence of TLR4 antagonist resatorvid (TAK-242, 1 μΜ; ApexBio, Houston, TX, USA), NF-κB inhibitor pyrrolidine dithiocarbamate (PDTC; 50 μM; Sigma), or combined with NF-κB activator phorbol myristate acetate (PMA, 100 ng/ml; Sigma), followed by treated with 30 μM BBR for 24 h.

### Quantitative real-time PCR (qRT-PCR)

Total RNA was extracted from treated podocytes with TRIzol reagent (Invitrogen Invitrogen, Carlsbad, CA, USA) and quantified by NanoDrop 2000/2000c spectrophotometer (Thermo Fisher Scientific, Waltham, MA, USA). Complementary DNA (cDNA) was synthesized from 1 µg total RNA by reverse transcription using a high capacity cDNA reverse transcription kit (TaKaRa, Tokyo, Japan). qPCR analysis of interleukin (IL)-1β, IL-6, and MCP-1 mRNA was performed with SYBR Premix ExTaq II kit (TaKaRa) and specific primers on an Applied Biosystems 7900 Real-Time PCR system (Applied Biosystems, Foster City, CA, USA). The relative quantification of mRNA levels was calculated based on the 2^−ΔΔCt^ method and normalized to GAPDH. The primers were as follows: GAPDH, forward: 5′-CAG TGC CAG CCT CGT CTA T-3′, reverse: 3′-AGG GGC CAT CCA CAG TCT TC-5′; IL-1β, forward: GTG ATG TTC CCA TTA GAC AGC, reverse: CTT TCA TCA CAC AGG ACA GG; IL-6, forward: 5′-ATG AAC TCC TTC TCC ACA AGC GC-3′, reverse: 5′-GAA GAG CCC TCA GGC TGG ACT G-3′; MCP-1, forward: 5′-TCA GCC AGA TGC AGT TAA CGC-3′, reverse: 5′-TGA TCC TCT TGT AGC TCT CCA GC-3′.

### Western blot analysis

Kidney homogenate and podocytes were collected and lysed in cell lysis buffer (Beyotime, Haimen, China) with protease inhibitor cocktail and phosphatase inhibitor (both from Sigma-Aldrich) for protein extraction. Equal amount of protein lysates (30 μg) were separated by 10% serum dodecyl sulfate-polyacrylamide gels (SDS-PAGE) and electrotransferred onto nitrocellulose (NC) membranes (Millipore, Billerica, MA, USA). After being blocked with 5% non-fat dry milk in PBS for 1 h, the membranes were probed with the primary antibodies against TLR4, phosphorylated-p65 (p-p65), p65, p-IκBα, IκBα, Cleaved Caspase-3, Bcl-2 and β-actin (all from Santa Cruz Biotechnology, Santa Cruz, CA) at 4 °C overnight, followed by incubated with a horseradish peroxidase-conjugated secondary antibody (Invitrogen) for 2 h at room temperature. Peroxidase-labeled protein bands were detected by enhanced chemiluminescence reagents (Millipore) and the protein intensity was quantified with Image-Pro Plus 6.0 software (Media Cybernetics, Rockville, MD, USA).

### Apoptosis analysis

Podocytes were double stained with FITC-Annexin V and propidium iodide (PI) from a FITC Annexin V Apoptosis Detection Kit I (BD Biosciences, San Jose, CA, USA). The apoptotic rats were analyzed using a FACScan flow cytometer (BD Biosciences).

### Statistical analysis

Data are displayed as mean ± standard deviation (SD). Statistical analysis was performed with GraphPad Prism 5 software (GraphPad Software Inc., San Diego, CA, USA). Comparison among experimental groups was performed using unpaired two-tailed Student’s *t* test and analysis of variance (ANOVA), with a value of *P* < 0.05 being considered as statistically significant.

## Results

### BBR ameliorated renal injury in STZ-induced DN rat model

**Fig. 1:**
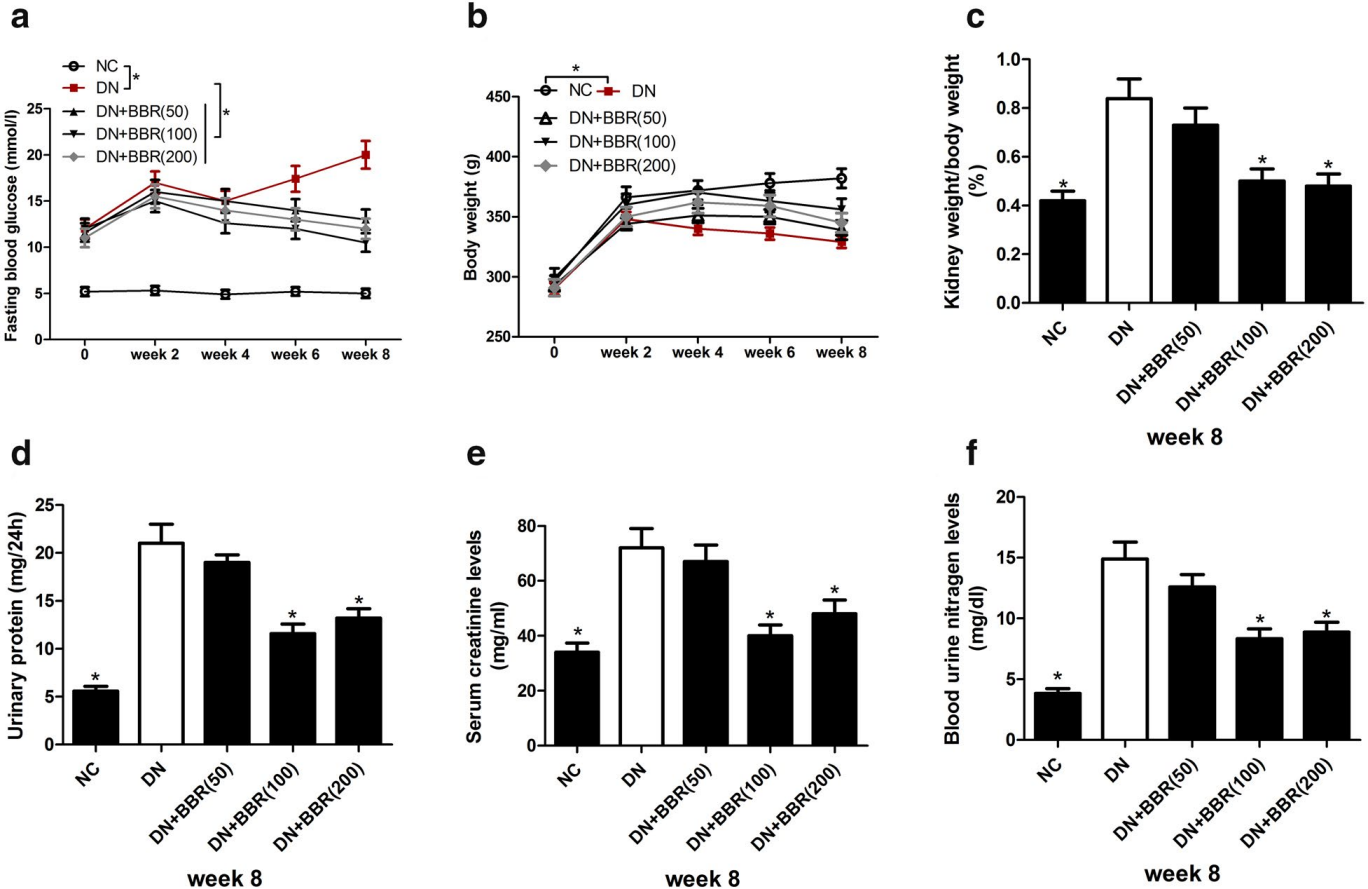
Effects of BBR on metabolic and biochemical parameters in STZ-induced DN rats. **a** Time course study of fasting blood glucose levels. **b** Time course study of body weight. **c** Ratio of kidney weight to body weight at 8 weeks after BBR treatment. **d** 24-h urinary protein at 8 weeks after BBR treatment. **e** Serum creatinine at 8 weeks after BBR treatment. **f** Blood urea nitrogen at 8 weeks after BBR treatment. *P < 0.05, n = 3

To determine the protective effect of BBR in DN, the indexes associated with kidney function, including fasting blood glucose levels, body weight, ratio of kidney weight to body weight, 24-h urinary protein, serum creatinine and blood urea nitrogen were measured. The results showed that STZ injection resulted in a significant increase in fasting blood glucose level (Fig. 1a), kidney/body weight (Fig. 1c), 24 h-urinary protein level (Fig. 1d), serum creatinine level (Fig. 1e) and blood urea nitrogen level (Fig. 1f) when compared with NC group. However, administration with BBR (100 or 200 mg/kg) significantly attenuated these effects in STZ-induced DN rats. Treatment with BBR at a low dose of 50 mg/kg exhibited no significant influence on these parameters in STZ-induced DN rats. Moreover, the body weight of STZ-induced DN rats was obviously decreased compared with DN group, while BBR treatment displayed little effect in restoring the lowered weigh (Fig. 1b). Collectively, these results demonstrated that BBR could ameliorate renal injury in STZ-induced DN rats.

### BBR attenuated the systemic and renal cortex inflammatory response in STZ-induced DN rats

**Fig. 2:**
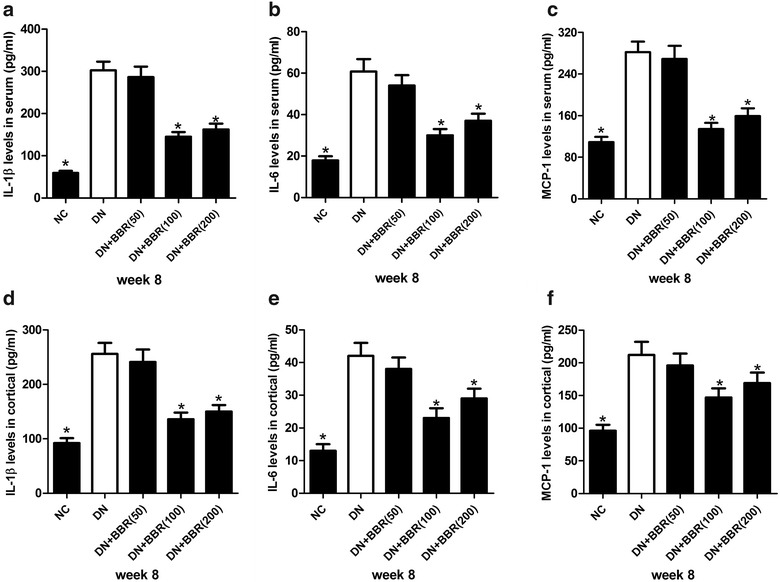
BBR ameliorated the inflammatory response in STZ-induced DN rats. The concentrations of inflammatory factors IL-1β (**a**, **d**), IL-6 (**b**, **e**), and MCP-1 (**c**, **f**) in serum and renal cortex of NC group, DN group, DN + BBR (50 mg/kg) group, DN + BBR (100 mg/kg) group, and DN + BBR (200 mg/kg) group were measured by ELISA. *P < 0.05, n = 3

It is well established that inflammation played an important role in the pathogenesis of DN. We therefore determined the effect of BBR on renal inflammation in DN rats by measuring the concentrations of inflammatory factors IL-1β, IL-6, and MCP-1 in the serum and renal cortex of rats. The ELISA results revealed that compared with untreated rats, an evident rise in the production of proinflammatory cytokines including IL-1β (Fig. 2a, d) and IL-6 (Fig. 2b, e), and chemokine MCP-1 (Fig. 2c, f) was observed in the serum and renal cortex of STZ-induced DN rats. However, oral administration with BBR (100 or 200 mg/kg) significantly inhibited the generation of IL-1β, IL-6 and MCP-1 in the serum (Fig. 2a–c) and renal cortex (Fig. 2d–f) of DN rats. BBR at a dose of 50 mg/kg had no apparent effect on inflammatory response of STZ-induced DN rat model. Therefore, we concluded that BBR attenuated the systemic and renal cortex inflammatory response in STZ-induced DN rats.

### BBR inhibited the activation of TLR4/NF-κB pathway in STZ-induced DN rat model

**Fig. 3:**
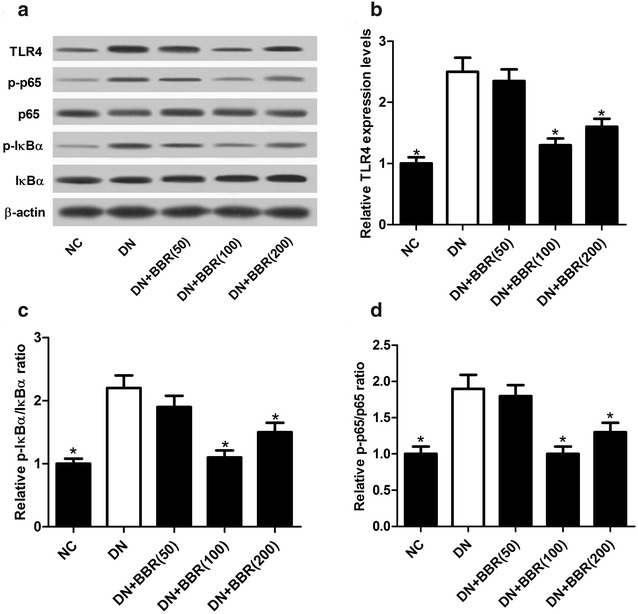
BBR inactivated TLR4/NF-κB pathway in STZ-induced DN rat model. **a**–**d** Western blot was used to detect the protein levels of TLR4, p-IκBα, IκBα, p-p65, and p65 in NC group, DN group, DN + BBR (50 mg/kg) group, DN + BBR (100 mg/kg) group, and DN + BBR (200 mg/kg) group. *P < 0.05, n = 3

Since the TLR4/NF-κB pathway has been extensively reported to be involved in inflammatory response, we therefore examined whether the protective effect of BBR in DN rats was associated with the TLR4/NF-κB pathway. We detected the protein levels of TLR4, NF-κB pathway key factors p65 and IκBα in DN rats after administration with BBR (50, 100 or 200 mg/kg) by western blot. The protein level of TLR4 (Fig. 3a, b), p-IκBα/IκBα ratio (Fig. 3a, c) and p-p65/p65 ratio (Fig. 3a, d) were markedly up-regulated in STZ-induced DN rat model compared to NC group, suggesting that the TLR4/NF-κB pathway was activated in STZ-induced DN rats. However, treatment with BBR (100 or 200 mg/kg) prominently restrained the activation of the TLR4/NF-κB pathway in DN rats by repressing the protein level of TLR4 and phophorylation of IκBα and p65 (Fig. 3a–d). Whereas, BBR (50 mg/kg) did not significantly suppress TLR4/NF-κB pathway in DN rats. Taken together, these data indicated that BBR blocked the activation of TLR4/NF-κB pathway in STZ-induced DN rats.

### BBR decreased HG-induced apoptosis of podocytes

**Fig. 4:**
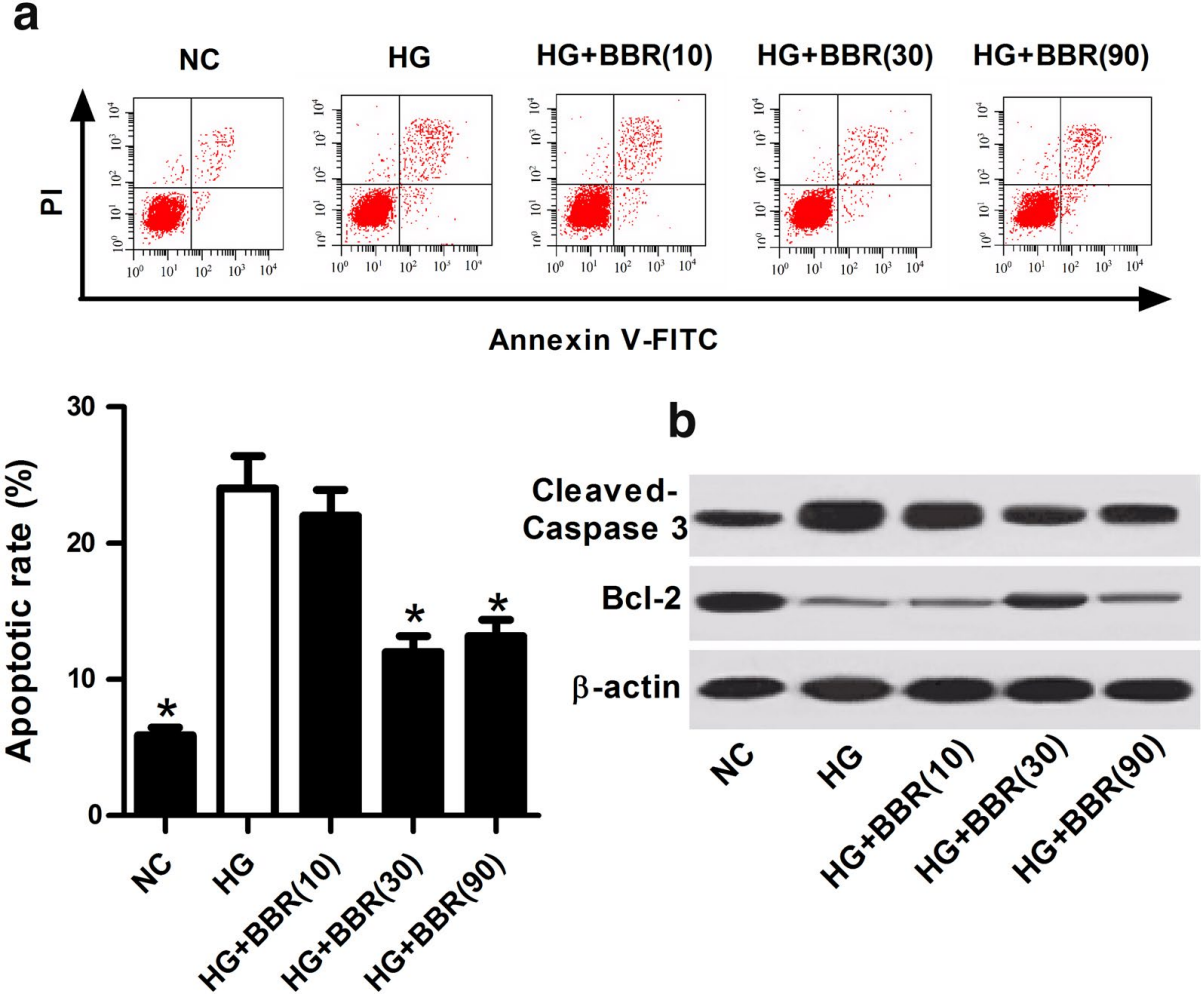
BBR repressed HG-induced apoptosis of podocytes. **a** Flow cytometry analysis was used to examine the apoptotic rats in HG-induced podocytes treated with 10, 30, or 90 μM BBR. **b** Western blot was performed to detect the protein levels of Cleaved Caspase-3 and Bcl-2 in HG-induced podocytes treated with 10, 30, or 90 μM BBR. *P < 0.05, n = 3

To determine the protective effect of BBR in DN in vitro, an in vitro DN model was established by treating podocytes with 30 mM high glucose for 24 h. We performed flow cytometry analysis to explore the effect of BBR on HG-induced apoptosis in podocytes. The results exhibited that HG treatment strikingly induced cell apoptosis relative to untreated podocytes, whereas BBR challenge at 30 or 90 μM notably attenuated HG-induced apoptosis of podocytes (Fig. 4a). Consistently, western blot analyses further demonstrated that HG exposure resulted in an obvious increase of Cleaved Caspase-3 and an evident decrease of Bcl-2 in podocytes, while these effects were significantly reversed by oral administration of BBR (Fig. 4b). These results suggested that BBR suppressed HG-induced apoptosis of podocytes.

### BBR weakened HG-induced inflammatory response in podocytes

**Fig. 5:**
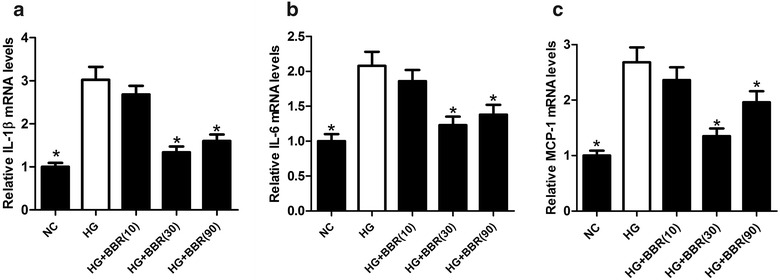
BBR mitigated HG-induced inflammatory response in podocytes. The mRNA amounts of IL-1β (**a**), IL-6 (**b**), and MCP-1 (**c**) in HG-induced podocytes treated with 10, 30, or 90 μM were estimated by qRT-PCR. *P < 0.05, n = 3

To investigate the effect of BBR on inflammatory response in DN in vitro, the mRNA amounts of inflammatory factors IL-1β, IL-6, and MCP-1 in HG-induced podocytes treated with 10, 30, or 90 μM were determined by qRT-PCR. Consistently with the in vivo results, the mRNA amounts of IL-1β (Fig. 5a), IL-6 (Fig. 5b), and MCP-1 (Fig. 5c) in HG-induced podocytes were conspicuously upregulated in comparison with NC group, while these effects were markedly abated following BBR treatment (30 or 90 μM), suggesting that BBR hindered HG-induced inflammatory response in podocytes.

### BBR blocked the activation of TLR4/NF-κB pathway in HG-induced podocytes

**Fig. 6:**
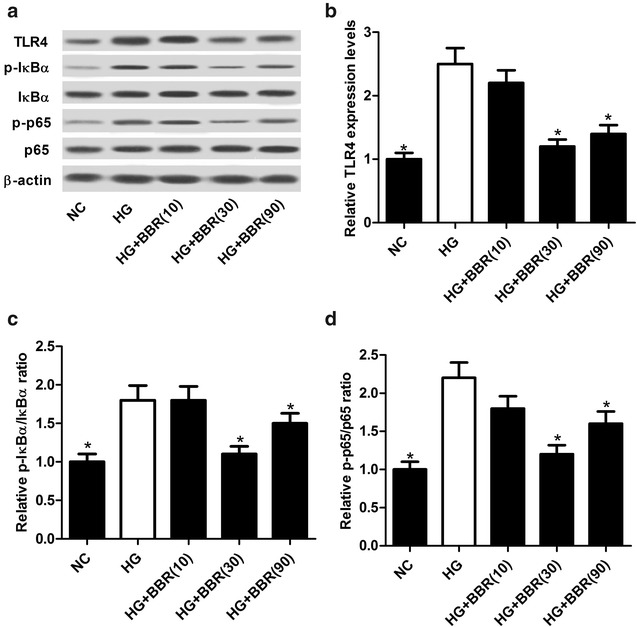
BBR impeded the activation of TLR4/NF-κB pathway in HG-induced podocytes. The protein levels of TLR4 (**a**, **b**), p-IκBα/IκBα ratio (**a**, **c**) and p-p65/p65 ratio (**a**, **d**) in HG-induced podocytes treated with 10, 30, or 90 μM were determined by western blot. *P < 0.05, n = 3

The effect of BBR on the TLR4/NF-κB pathway in HG-induced podocytes was further analyzed. Western blot analyses demonstrated that the protein levels of TLR4 (Fig. 6a, b), p-IκBα/IκBα ratio (Fig. 6a, c) and p-p65/p65 ratio (Fig. 6a, d) were all elevated in HG-treated podocytes, while these effects were markedly attenuated following BBR treatment (30 or 90 μM), indicating that BBR inactivated the TLR4/NF-κB pathway in HG-induced podocytes.

### BBR treatment inhibited HG-induced inflammatory response and apoptosis in podocytes by blocking TLR4/NF-κB pathway

**Fig. 7:**
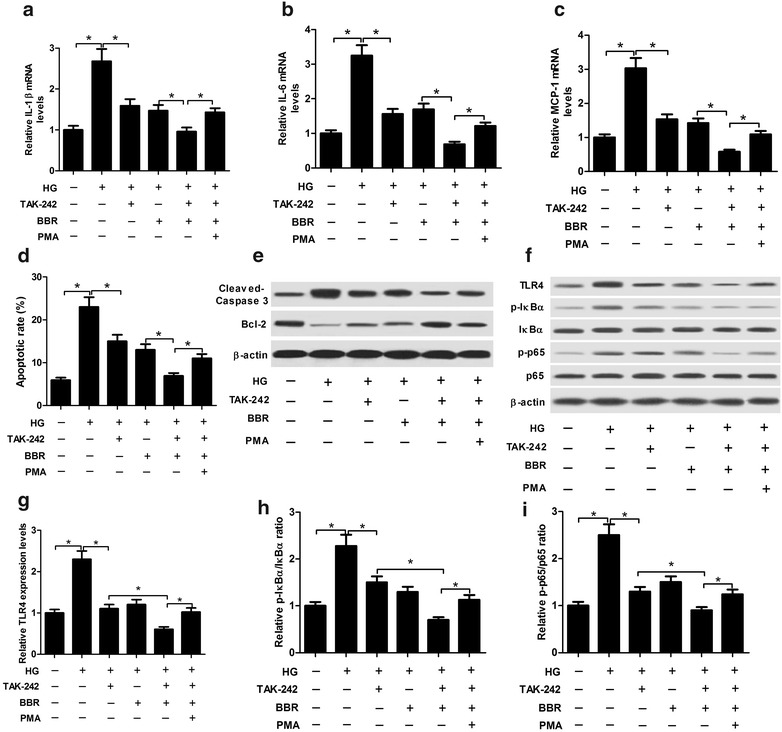
Effects of TAK-242, or combined with BBR or/and PMA on inflammatory response and apoptosis in HG-induced podocytes. Podocytes were pre-treated with or without 1 μΜ TAK-242, or along with 100 ng/ml PMA for 2 h prior to stimulating with 30 mM high glucose. Then, cells were treated with 30 μM BBR for 24 h. The mRNA amounts of IL-1β (**a**), IL-6 (**b**), and MCP-1 (**c**) in treated podocytes were measured by western blot. **d** Flow cytometry analysis was performed to detect apoptotic rates of treated podocytes. **e** The protein levels of Cleaved Caspase-3 and Bcl-2 in treated podocytes were determined by western blot. **f**–**i** The protein levels of TLR4, p-IκBα, IκBα, p-p65, p65 in treated podocytes were determined by western blot. *P < 0.05, n = 3

TAK-242, a TLR4 inhibitor, was employed to explore the underlying mechanism of BBR in DN in vitro. We selected the BBR at a dose of 30 μΜ for further analysis based on the above results. Podocytes were stimulated with 30 mM high glucose in the presence or absence of 1 μΜ TAK-242, followed by treated with 30 μM BBR for 24 h. qRT-PCR results demonstrated that TAK-242 treatment prominently repressed HG-induced upregulation of inflammatory factors including IL-1β (Fig. 7a), IL-6 (Fig. 7b), and MCP-1 (Fig. 7c) in podocytes. Moreover, cotreatment with BBR and TAK-242 aggravated BBR-mediated inhibition on the amounts of IL-1β, IL-6, and MCP-1 in HG-induced podocytes, which was partially abated following the addition of PMA (Fig. 7a–c). Meanwhile, flow cytometry analysis demonstrated that TAK-242 administration significantly lowered HG-induced apoptosis in podocytes, while BBR treatment significantly intensified BBR-induced anti-apoptotic effect in HG-treated podocytes, which was considerably restored by the treatment of PMA (Fig. 7d). Consistently, Western blot analyses demonstrated that TAK-242 administration significantly reduced Cleaved Caspase-3 protein level and significantly increased Bcl-2 protein level in HG-treated podocytes, while cotreatment with TAK-242 and BBR markedly overturned TAK-242-induced decrease of Cleaved Caspase-3 level and increase of Bcl-2 level, which was further reversed following the addition of PMA (Fig. 7e). Additionally, we found that TAK-242 treatment significantly suppressed the TLR4/NF-κB pathway in HG-induced podocytes by reducing the protein level of TLR4 (Fig. 7f, g), p-IκBα/IκBα ratio (Fig. 7f, h) and p-p65/p65 ratio (Fig. 7f, i). Moreover, the inhibitory effect on TLR4/NF-κB elicited by BBR challenge was exacerbated following treatment with TAK-242 in HG-induced podocytes, which was distinctly overturned by PMA treatment. Collectively, these findings demonstrated that BBR treatment inhibited HG-induced inflammatory response and apoptosis in podocytes by blocking TLR4/NF-κB pathway.

### PDTC treatment aggravated the inhibitory effect of BBR on HG-induced inflammatory response and apoptosis in podocytes

**Fig. 8:**
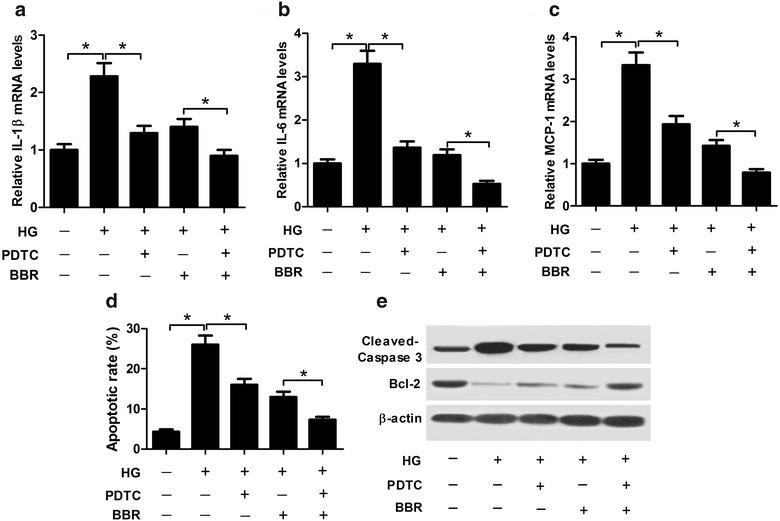
BBR relieved HG-induced inflammatory response and apoptosis in podocytes by hindering TLR4/NF-κB pathway. Podocytes were pre-treated with or without 50 μΜ PDTC for 2 h prior to stimulating with 30 mM high glucose, followed by treated with 30 μM BBR for 24 h. The mRNA amounts of IL-1β (**a**), IL-6 (**b**), and MCP-1 (**c**) in treated podocytes were assessed by western blot. **d** The apoptotic rates of treated podocytes were measured by flow cytometry analysis. **e** Western blot analysis of the protein levels of Cleaved Caspase-3 and Bcl-2. *P < 0.05, n = 3

PDTC, a NF-κB inhibitor, was further used to confirm whether the inhibitory effect of BBR on HG-induced inflammatory response and apoptosis in podocytes was mediated by TLR4/NF-κB pathway. Podocytes were pre-treated with or without 50 μΜ PDTC for 2 h prior to stimulating with 30 mM high glucose. Then, cells were exposed to 30 μM BBR for 24 h. qRT-PCR results demonstrated that inactivation of TLR4/NF-κB pathway by PDTC significantly reduced HG-induced increase in the amounts of IL-1β (Fig. 8a), IL-6 (Fig. 8b), MCP-1 (Fig. 8c), as well as apoptosis (Fig. 8d) in podocytes. Furthermore, simultaneous PDTC and BBR treatment significantly aggravated the inhibitory effect of BBR on inflammatory factors amount and cell apoptosis in HG-treated podocytes. Additionally, western blot demonstrated that PDTC treatment significantly reduced the protein level of Cleaved Caspase-3 and greatly enhanced Bcl-2 amount in HG-treated podocytes, which was substantially reversed following the addition of BBR (Fig. 8e). These results suggested that BBR blocked HG-induced inflammatory response and apoptosis in podocytes by suppressing TLR4/NF-κB pathway.

## Discussion

The current study evaluated the renoprotective effects and molecular mechanism of BBR on the pathogenesis of DN in STZ-induced DN rat model and HG-induced DN podocytes model. In the present study, we provided strong evidence that BBR not only attenuated renal dysfunction and inflammatory response, but also suppressed TLR4/NF-κB pathway in STZ-induced DN rats and HG-induced podocytes. Furthermore, mechanistic analysis demonstrated that blockade of TLR4/NF-κB pathway by TAK-242 or PDTC exacerbated the inhibitory effect of BBR on HG-induced inflammatory response and apoptosis in podocytes. Therefore, our study suggested that BBR ameliorated DN by inhibiting TLR4/NF-κB pathway.

As has been well described, BBR has a wide range of pharmacological activities and has been demonstrated to exert protective effects against DN progression by ameliorating a variety of pathological changes [22]. More recently, it was demonstrated that BBR displayed a tendency to ameliorate renal dysfunction and renal inflammation, improve glucose and lipid metabolism disorders, and decrease fasting blood glucose in DN rats [23, 24]. Moreover, BBR could inhibit renal fibrosis and inhibited renal tubular epithelial-to-mesenchymal transition of DN [25, 26]. Additionally, BBR inhibits high glucose-induced glomerular mesangial cell proliferation and ECM accumulation, and attenuates tubulointerstitial fibrosis in DN, which suggests that BBR can be used as a further potential therapeutic drug for DN [27, 28]. Additionally, it was previously demonstrated that the doses of BBR that can be administrated in patients with type 2 diabetes and diabetic rats are 300 mg three times a day and 100 mg/kg, respectively [29, 30]. In the present study, we demonstrated that BBR challenge at 100 or 200 mg/kg significantly reduced renal injury in STZ-induced DN rat model, as evidenced by a decrease in fasting blood glucose, ratio of kidney weight to body weight, 24-h urinary protein, serum creatinine, and blood urine nitrogen, while BBR at a low dose of 50 mg/kg had no significant effect on these parameters associated with kidney function. Moreover, we found that BBR also weakened STZ-induced inflammatory factors production in DN rats, and inhibited HG-induced apoptosis and inflammatory response in podocytes, confirming the renoprotective effect of BBR in DN.

There is increasing evidence supporting that inflammation exerts important roles in the pathogenesis of DN [31]. TLR4/NF-κB pathway appears to play a critical role in the pathogenesis with a variety of inflammatory conditions [32]. The proinflammatory role of TLR4/NF-κB pathway has been demonstrated to be involved in the progression of diabetes and diabetic complication [33, 34]. Upon stimulation, activation of TLR4 pathway subsequently activated the NF-κB pathway and triggered NF-κB-dependent inflammatory response, which might ultimately aggravate renal dysfunction in acute and chronic kidney diseases [35, 36]. In podocytes and tubular epithelial cells, exposure to high glucose promoted TLR4 activation, resulting in NF-κB activation and consequent inflammatory and fibrogenic responses [37]. Silencing of TLR4 with small interfering RNA attenuated high glucose-induced IκB/NF-κB activation and inhibited downstream inflammatory cytokines IL-6 and chemokine (C–C motif) ligand 2 (CCL-2) in human proximal tubular epithelial cells [16]. Consistently, the present study demonstrated that BBR treatment blocked HG-induced activation of TLR4/NF-κB pathway in both STZ-induced DN rats and HG-treated podocytes. It was previously reported that BBR inhibited the NF-κB activation and fibronectin (FN) accumulation by inhibiting RhoA/ROCK signaling in diabetic rat kidneys and high glucose-induced glomerular mesangial cells [23]. In addition, BBR could improve insulin resistance of skeletal muscle through inhibiting the active of the TLR4/IκBβ/NF-κB signaling pathway [38]. BBR inhibited IL-1β-induced nitric oxide (NO) production in primary mixed glia and BV-2 cells via inactivation of TLR4/adapter protein myeloid differentiation factor 88 (MyD88)/NF-κB signaling [39]. Besides, the anti-bacterial effects of BBR were mediated by acting as a high-affinity LPS antagonist and blocking the LPS/TLR4 signaling [40]. Moreover, BBR repressed LPS-induced cell proliferation and FN expression in rat mesangial cells through impeding the activation of NF-κB signaling pathway and protein expression of its downstream inflammatory mediators [26]. Our study further revealed that inhibition of TLR4/NF-κB pathway by TAK-242 or PDTC suppressed the HG-induced inflammatory response and apoptosis in podocytes. Moreover, combination with BBR treatment and inhibition of TLR4/NF-κB pathway exacerbated the inhibitory effect of BBR on HG-induced inflammatory response and apoptosis in podocytes, indicating that BBR ameliorated DN by inhibiting the TLR4/NF-κB pathway. Additionally, many other signaling pathways, such as advanced glycation end products-receptor for AGEs (AGEs-RAGE) [14] and AMP-activated protein kinase (AMPK) [41], have been elucidated to be implicated in the renoprotective effects induced by BBR.

## Conclusions

In conclusion, our study demonstrated that BBR ameliorated DN by attenuating renal injury, inflammatory response and podocytes apoptosis. Mechanistic analysis revealed that the renoprotective effect of BBR on DN depended on inhibition of TLR4/NF-κB pathway. The present study contributed to a better understanding of the mechanism underlying BBR involved in DN and provided new evidence for the potential application of BBR in the treatment of DN.

## Abbreviations

DN: diabetic nephropathy
PDTC: pyrrolidine dithiocarbamate
ECM: extracellular matrix
TGF-β1: transforming growth factor-β1
TNF-α: tumor necrosis factor-α
BBR: berberine
TLRs: toll-like receptors
NF-κB: nuclear factor kappa B
MCP-1: monocyte chemotactic protein-1
STZ: streptozotocin
ELISA: enzyme-linked immunosorbent assay
SDS-PAGE: serum dodecyl sulfate-polyacrylamide gels
PI: propidium iodide
HG: high glucose
TAK-242: resatorvid
IL: interleukin
IFNy: interferon-y
cDNA: complementary DNA
NC: nitrocellulose
CCL-2: chemokine (C–C motif) ligand 2
FN: fibronectin
AGEs-RAGE: advanced glycation end products-receptor for AGEs
AMPK: AMP-activated protein kinase
